# Editorial: Progressive keratoconus: insights into etiopathogenesis, diagnosis, and treatment

**DOI:** 10.3389/fmed.2023.1302625

**Published:** 2023-11-13

**Authors:** Cristina Ariadna Nicula, Adriana Elena Bulboaca

**Affiliations:** ^1^Department of Maxi-lo-Facial Surgery and Radiology, Dental Implantology, “Iuliu Hatieganu” University of Medicine and Pharmacy, Cluj-Napoca, Romania; ^2^“Iuliu Hatieganu” University of Medicine and Pharmacy, Cluj-Napoca, Romania

**Keywords:** keratoconus (KCN), cross-linking, KCN treatment, KCN progression, corneal tomography

Keratoconus (KCN) is a bilateral and asymmetric corneal ectatic dystrophy with onset during the second decade of life. The development of modern topographic, tomographic, and biomechanical examinations has increased the accuracy of keratoconus diagnosis, particularly in the subclinical stages of the disease. Steinwender et al. analyzed deviations between the locations of different tomographic parameters and identified low consistency between different methods for describing the location of a keratoconus. This study suggested that elevation- or pachymetry-based measures (such as ELEB, ELEF, or Pachymin) are suitable for clinical applications requiring an accurate cone center (Steinwender et al.). Furthermore, Mazharian et al. suggested that a significant proportion of KCN patients are likely to remain stable if close monitoring and strict eye rubbing cessation are achieved, without the need for further intervention. Moreover, Zhao X. et al. analyzed the levels of sex hormones associated with KCN and revealed that testosterone levels were reduced in the plasma of both female and male KCN patients, whereas plasma estradiol levels were increased in only male KCN patients. Moreover, when analyzing tear samples from KCN patients, Goñi et al. found A-kinase anchor protein 13 among the deregulated proteins detected, deserving special attention for its involvement in corneal thinning and its strong overexpression in the tears of patients with more active KC and faster disease progression.

In recent decades, cross-linking therapy has been successfully applied to stop or arrest KCN. Wajnsztajn et al. studied the best outcome indicators for cross-linking in pediatric KCN (logMAR visual acuity, maximal corneal power, pachymetry, and refractive cylinder). They concluded that non-accelerated treatment was more effective than accelerated treatment and that corneas with advanced disease had a greater effect on CXL (Wajnsztajn et al.). Moreover, Liu et al. demonstrated the application of a machine-learning approach using the XGBoost model, which produced predicted values closest to the actual values for both the CDVA and maximal corneal power changes in the testing (R2 = 0.9993 and 0.9888) and validation (R2 = 0.8956 and 0.8382) sets. Kobashi et al. suggested an experimental model using collagenase treatment to induce KCN by steepening the keratometric and astigmatic values. This study identified no significant difference in the observed elastic behaviors of normal and ectatic corneas under physiologically relevant stress levels. Moreover, ultraviolet irradiation did not cause the regression of corneal steepening in a collagenase-induced model during short-term observation (Kobashi et al.). Mazzotta et al. introduced a novel cross-linking technique, the higher-fluence pulsed light Epi-ON accelerated crosslinking nomogram (PFPL M Epi-On ACXL), for the treatment of progressive KCN. A non-randomized interventional study showed that the pachymetry-based PFPL M Epi-On ACXL nomogram stabilized ectasia progression and higher fluence Epi-On ACXL increased CXL penetration with better functional outcomes in the absence of complications. Furthermore, the coma value improved significantly by the 6 month in all groups (Mazzotta et al.). Bonzano et al. investigated the demographic and corneal factors associated with the occurrence of delayed re-epithelialization after epithelium-off CXL (epi-off CXL) and concluded that the association between delayed re-epithelialization and age may reflect an age-related decrease in the corneal healing response without changing the efficacy of cross-linking.

Zhao Z. et al. showed that voriconazole combined with cross-linking was effective in treating Aspergillus-infected keratitis. They concluded that combination therapy could effectively inhibit Aspergillus, accelerate corneal repair, and shorten the disease course (Zhao Z. et al.).

## Author contributions

CN: Writing—original draft, Writing—review & editing. AB: Writing—original draft, Writing—review & editing.

